# Effects of RyhB on Fur regulation of cytochrome *c* synthesis in *Shewanella oneidensis*

**DOI:** 10.1128/aem.01500-26

**Published:** 2026-09-30

**Authors:** Kaiyue Jie, Xinyue Liu, Fuqian Zhang, Jim Yah, Lulu Liu, Haichun Gao

**Affiliations:** 1 State Key Laboratory for Vegetation Structure, Function and Construction (VegLab), Institute of Microbiology, College of Life Sciences, Zhejiang University 98445, Hangzhou, China; 2 Sir William Dunn School of Pathology, University of Oxford 6396, Oxford, United Kingdom; Indiana University Bloomington, Bloomington, Indiana, USA

**Keywords:** iron homeostasis, RyhB, cytochrome *c*, sRNA, Fur

## Abstract

*Shewanella oneidensis* is one of the most extensively studied electrochemically active bacteria because of its unparalleled respiratory versatility, which is largely built on diverse cytochromes (cyts) *c*. It has been firmly established that Fur, RyhB, and Hfq are critical for cyt *c* production through regulation of iron homeostasis, but how they interplay remains largely unknown. In this study, we show that *S. oneidensis* RyhB carries novel features, such as unusual length and functional exchangeability with its *Escherichia coli* counterpart but not its *Vibrio cholerae* counterpart. The RyhB loss suppresses the signature phenotypes caused by the Fur loss, including impaired growth and cyt *c* production in *S. oneidensis*. Despite this, neither the direct regulation of expression of most cyt *c* genes nor the suppression of disrupted iron homeostasis resulting from Fur loss appears to be a primary mechanism underlying RyhB’s influence on cyt *c* content. Rather, this effect is achieved by modulating heme levels; RyhB mediates the expression of the key genes in heme biosynthesis and the entire heme uptake/degradation system. Overall, our work highlights the significance of RyhB-mediated post-transcriptional regulation in the physiology of *S. oneidensis*, unraveling unexpected mechanisms by which RyhB affects cyt *c* biosynthesis.

**IMPORTANCE:**

In bacteria, RyhB has been implicated in the regulation of diverse biological processes post-translationally. In *Shewanella oneidensis*, both Fur and RyhB influence the cytochrome (cyt) *c* content, which are the cornerstones of bacterial respiratory versatility and potential application in bioenergy and bioremediation. Here, we show that while RyhB generally exhibits an antagonistic relationship with Fur, it carries novel sequence features and differentially regulates heme biosynthesis and degradation, leading to altered cyt *c* content. The data presented show that the biological processes in a given bacterium regulated by RyhB are highly complex, amounting to the required coordination of multiple regulators to allow cells to respond to environmental changes promptly.

## INTRODUCTION

Iron is an essential metal for virtually all living organisms because it primarily functions as a versatile prosthetic component for proteins that are mostly irreplaceable in an array of biological processes, such as respiration, DNA synthesis, and metabolism (1). When overloaded, however, iron is highly toxic to the cell by leading to oxidative stress, which damages the chromosome, lipid membranes, and redox proteins (2). Accordingly, the dynamics of intracellular iron concentrations, termed iron homeostasis, must be precisely controlled by balancing uptake, storage, utilization, and export (3).

In bacteria, the regulation of iron homeostasis is carried out by an intricate network consisting of a variety of regulators that function at multiple levels through sophisticated mechanisms (3). At the transcription level, many regulators that sense intracellular iron concentrations and modulate gene expression have been identified. Among them, Fur (ferrous uptake regulator), which has been most extensively studied in *Escherichia coli*, represents the classical model of transcriptional repression by binding to the Fur box when bound to the co-repressor Fe^2+^ (4). When iron is limited, Fur dissociates from the metal co-repressor and falls off the Fur box, allowing transcription of the genes that follow. Apart from this, Fur may function through other regulatory modes, including transcriptional activation by the metalated regulator and repression by the apo-protein (5, 6). Iron homeostasis is also subjected to post-transcriptional regulation by a number of small RNAs (sRNAs), RyhB in particular, along with RNA chaperones, such as Hfq. By base pairing with target mRNAs or interacting with proteins, they influence translation, stability, and degradation of transcripts (7).

*Shewanella* comprise a large group of facultative anaerobic γ-proteobacteria that are renowned for remarkable respiratory versatility and hold significant promise in bioenergy, including applications in microbial fuel cells, bioelectrochemical systems, and biohydrogen production (8). To support this versatility, these bacteria generally host a vast number of iron-containing proteins, especially cytochrome *c* (cyt *c*) proteins, a type of hemoprotein that carries covalently attached heme and is primarily involved in extracellular electron transport and/or oxidoreduction (9, 10). In the extensively studied representative of the genus, *Shewanella oneidensis*, more than 40 distinct cyt *c* proteins are encoded. As a result, *S. oneidensis* has iron demand substantially higher than model organisms, such as *E. coli*, and therefore mandates tight regulation of iron homeostasis (11–15).

In *S. oneidensis*, all of Fur, sRNA RyhB, and RNA chaperone Hfq are implicated in regulating the cyt *c* content, either directly or indirectly. Although Fur directly mediates transcription of a few cyt *c* genes, it is the disrupted iron homeostasis that leads to significantly reduced cyt *c* content upon Fur loss (12, 16, 17). RyhB was demonstrated to directly repress the expression of a couple of cyt *c* genes, and an *ryhB* null mutant was found to have elevated cyt *c* content (18). In contrast, Hfq was also shown to affect the cyt *c* content in an indirect manner, but the effects reported to date are not consistent (18–20).

Given that cyt *c* proteins are central to the respiratory versatility and potential application of *S. oneidensis*, further investigations into the physiological impacts of Fur and RyhB, and more importantly, their interplay, are much needed. In this study, we systematically examined the physiological influences of RyhB on cyt *c* content and how this sRNA interplays with Fur to maintain iron homeostasis.

## RESULTS

### The loss of RyhB largely suppresses the defect of the *fur* mutant in growth and cyt *c* content

**Fig 1:**
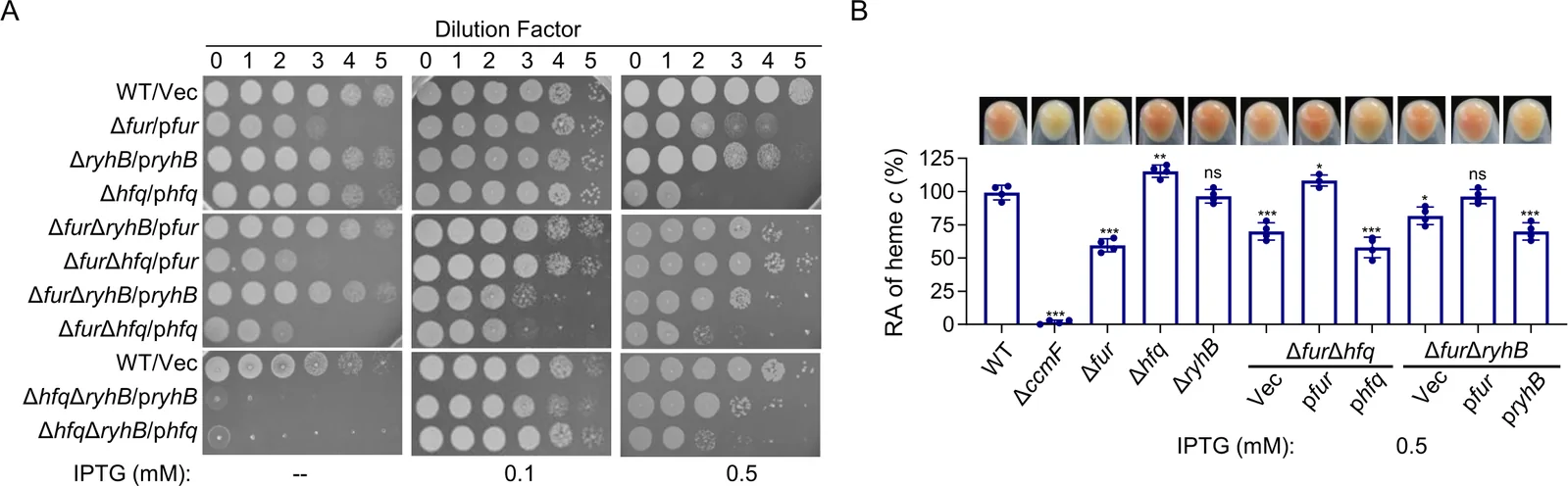
Physiological impacts of loss of Fur, Hfq, and RyhB in *S. oneidensis*. (**A**) Growth (plating efficiency) of the *fur, hfq,* and *ryhB* single and double mutants on LB plates. Cultures grown to the mid-log phase (OD_600_ ≈ 0.4) were adjusted to contain approximately 10^8^ CFU/mL as the undiluted cultures (dilution factor, 0), which were then subjected to 10-fold serial dilutions. Five microliters of each dilution was dropped onto agar plates, and the results were photographed 18 h later. WT, wild-type strain; Vec, carrying an empty vector; and p*fur*, expressing a copy of *fur* on a plasmid in *trans* for complementation and the same was for other genes. Gene expression was driven by the IPTG-inducible promoter P*tac*. (**B**) Cell pellets and cyt *c* contents in the strains under test grown to the early stationary phase. The heme *c* levels of the WT and cyt *c*-deficient strain ∆*ccmF* were set to 1 and 0, respectively. Relative abundance of heme *c* in each strain under test was given by normalizing to the average of the WT levels. In panel A, experiments were independently performed at least four times, and representative data are presented. In panel B, the data from four replicates are presented as the mean ± SD. Statistical analysis was performed against the WT data: ns, not significant; *, *P* < 0.05; **, *P* < 0.01; and ***, *P* < 0.001.

It has been previously shown that the deletion of the *fur* gene (Δ*fur*) in *S. oneidensis* results in disrupted iron homeostasis, leading to impaired growth and light-colored cell pellets because of a significantly lowered cyt *c* content, compared to the wild type (WT) (12, 15). In contrast, the *hfq* deletion mutant (Δ*hfq*) grew comparably to the WT and has a modestly elevated cyt *c* content (by ~15%) (20). The data presented here are largely consistent with the reported phenotypes (Fig. 1A and B; Fig. S1A).

To explore the physiological impacts of RyhB in *S. oneidensis*, we attempted to delete *ryhB* from the WT, Δ*fur*, and Δ*hfq* strains. The impacts of the *ryhB* single deletion (Δ*ryhB*) on either growth or the cyt *c* content were insignificant (Fig. 1A and B; Fig. S1A through C). RyhB in excess (with 0.5 mM isopropyl-β-D-thiogalactopyranoside [IPTG] for induction), when expressed in *trans* from a vector under the control of IPTG, as demonstrated by Northern blotting (Fig. S1D), inhibited growth and lowered cyt *c* content (Fig. 1A; Fig. S1B and C). The effect of the RyhB loss became evident in the absence of Fur; the additional RyhB loss nearly fully suppressed the defects in both growth and the cyt *c* content of the Δ*fur* strain (Fig. 1A and B). In contrast, the removal of *hfq* from the Fur-absence background did not show detectable effects on growth or the cyt *c* content, implying that RyhB-based regulation is not fully dependent on Hfq. The changes in the cyt *c* content caused by these mutations were substantiated with heme staining (Fig. S1E).

Interestingly, while Δ*ryhB* and the double knockout strains Δ*fur*Δ*ryhB* were constructed smoothly, we failed to obtain Δ*hfq*Δ*ryhB*. The synthetic lethality of Hfq and RyhB was then confirmed by removing the *hfq* gene from the chromosome when a copy of *hfq* or *ryhB* was expressed in *trans* under the control of IPTG (Fig. 1A). The survival and growth of the Δ*hfq*Δ*ryhB*/p*hfq* and Δ*hfq*Δ*ryhB*/p*ryhB* strains were observed only in the presence of IPTG (Fig. 1A). The unexpected synthetic lethality rules out the possibility that RyhB is fully dependent on Hfq for its physiological role.

### Fur represses *ryhB* transcription, and Hfq promotes the stability of the *ryhB* transcript

**Fig 2:**
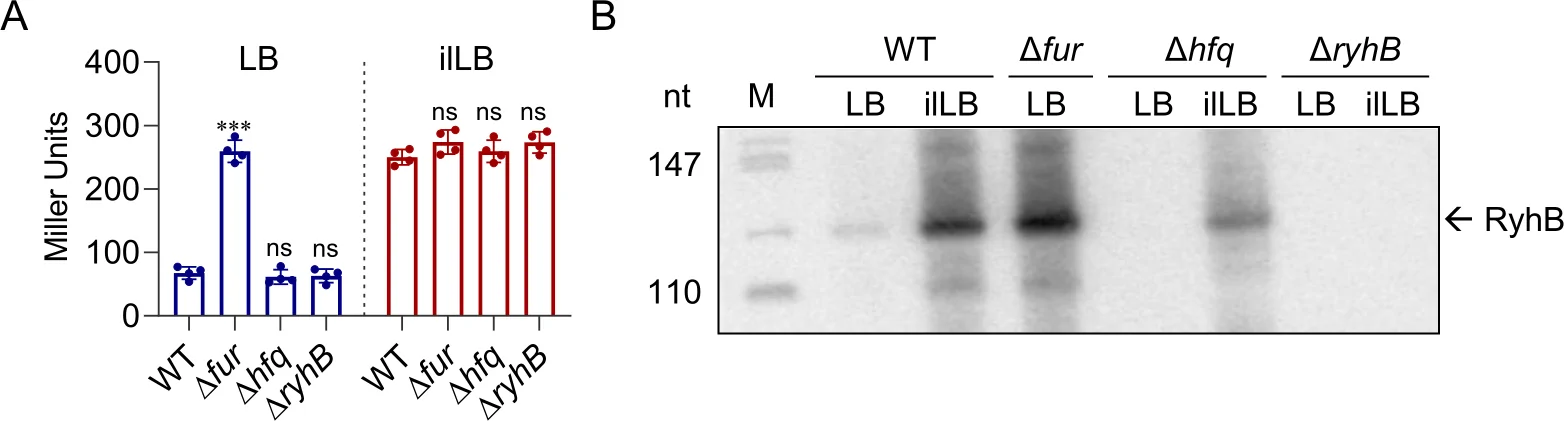
Impacts of Fur and Hfq on the expression of *ryhB*. (**A**) Expression of *ryhB* reflected by its promoter activity in indicated strains under different iron conditions by an integrative P*_ryhB_*-LacZ reporter. Cells of the mid-exponential phase (OD_600_ ≈ 0.4) were collected for β-galactosidase activity assay. ilLB, iron-limited LB. (**B**) Northern blotting analysis of RyhB. The same samples used in panel A were subjected to Northern blotting assay. M, marker. The experiment was independently performed at least four times. The data from four replicates and a representative are presented as the mean ± SD in panels A and B, respectively. Statistical analysis was performed against the average of the WT samples: ns, not significant; *, *P* < 0.05; **, *P* < 0.01; and ***, *P* < 0.001.

Given that the RyhB loss suppresses the physiological defects of Δ*fur*, this study primarily focuses on unraveling the mechanism underlying the suppression. To this end, we first monitored the impacts of iron levels and Fur loss on transcription of *ryhB*. Activity of the *ryhB* promoter was quantified in WT, Δ*fur*, Δ*ryhB*, and Δ*hfq* cells grown in lysogeny broth Lennox (LB) and iron-limited LB (ilLB) with an integrative LacZ reporter system, which was inserted into a degenerated gene far away from the original *ryhB* genomic locus. The ilLB medium was created by supplementing LB with 60 μM desferrioxamine (DFO), a commercially available siderophore that cannot be imported into *S. oneidensis* cells (13, 16). The promoter activity of the *ryhB* gene was very low in the WT cells grown in LB but increased more than four times when iron was depleted (Fig. 2A). A similar result was obtained from both the Δ*hfq* and Δ*ryhB* strains (Fig. 2A), suggesting that *ryhB* transcription is subjected to regulation by neither Hfq nor RyhB. In the *fur* mutant, however, the *ryhB* promoter was highly active in LB (Fig. 2A), resembling that in the WT grown in ilLB, indicating that Fur functions as a repressor of *ryhB* transcription.

By using Northern blotting, we found that the *ryhB* transcripts were not detected in Δ*ryhB* cells grown under normal or iron-limited conditions (Fig. 2B), verifying the genuineness of the mutant. Apparently, the abundance of *ryhB* transcripts in the WT and Δ*fur* strains matched the *ryhB* promoter activities as revealed above (Fig. 2A), supporting that *ryhB* expression is induced by iron starvation and repressed by Fur. However, the *ryhB* transcripts in Δ*hfq* were substantially less abundant than those in the WT strain grown under the same conditions (Fig. 2B). It should be mentioned that the amounts of RNA for all samples were quantified and adjusted to comparable levels, but the loading control was not included in the gel. As this result is in sharp contrast to the comparable activities of the *ryhB* promoter between these two strains, Hfq is likely able to promote the stability of the *ryhB* transcript without interfering with transcription.

### RyhB of *S. oneidensis* is distinct in terms of size and likely structure

**Fig 3:**
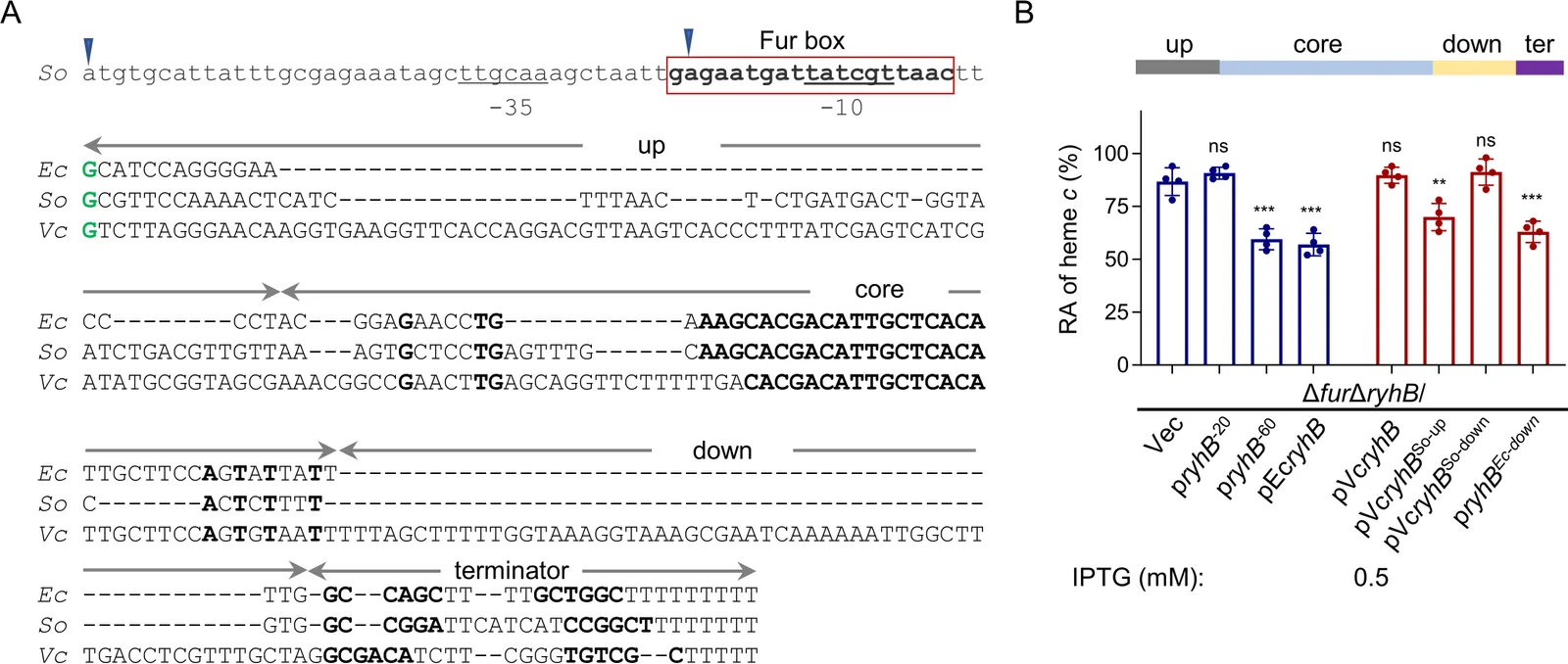
Sequence features of the *ryhB* locus in *S. oneidensis*. (**A**) Organization of *S. oneidensis ryhB* and alignment with the *E. coli and V. cholerae* counterparts. The promoter region, including the −35 and a −10 sequences (underlined), in the sequence (in lowercase) upstream of the coding sequence (in uppercase) is shown with the Fur box marked. The coding sequence consists of four segments: up, core, down, and terminator (ter), as shown in panel B, as predicted by sequence analysis, and the transcriptional start site (G, in green) was confirmed by 5′ RACE. The vertical arrowheads mark −60 and −20 nt nucleotide positions relative to the start, which delineate the sequences used for functional assessment in panel B. Nucleotides in bold in the core region and ter region represent the highly conserved and terminator palindromic sequences, respectively. (**B**) RyhB activity reflected by its impacts on cyt *c* levels. *VcryhB^So-up^* represents a *VcryhB* variant whose upstream sequence is replaced by the *SoryhB* counterpart. The heme *c* levels of the WT and cytochrome *c*-deficient strain ∆*ccmF* were set to 1 and 0, respectively. In panel B, the data from four replicates are presented as the mean ± SD. Statistical analysis was performed against the average of the Δ*fur*Δ*ryhB*/vec samples: ns, not significant; *, *P* < 0.05; **, *P* < 0.01; and ***, *P* < 0.001.

The Northern blot revealed that the transcript of *S. oneidensis ryhB* is 127 nt in length, significantly longer and shorter than its *E. coli* and *Vibrio cholerae* counterparts, which are 90 and 200+ nt, respectively (21, 22) (Fig. 3A). These *ryhB* sequences share a highly conserved core region, before and after which are two highly variable regions, namely RyhB^up^ and RyhB^down^, followed by the terminator region (Fig. 3A). Transcription of *S. oneidensis ryhB* is driven by a canonical σ^70^ promoter with highly conserved −35 and −10 boxes, and its start site was determined by 5′ RACE to be three nucleotides after the Fur box (12, 23) (Fig. 3A). These sequence features were then subjected to verification by expressing *S. oneidensis ryhB* of various lengths. The suppressing activity of the RyhB loss in the Δ*fur* strain was not affected by the expression of a 190-bp DNA fragment (*ryhB*^−20^) starting from 20 bp upstream of the transcription start site to the end of the gene (Fig. 3B), an expected result due to the absence of the −35 box of the promoter. In contrast, when a DNA fragment (*ryhB*^−60^) starting from 60 bp upstream of the transcription start site covers the intact promoter, the suppressing effect vanishes (Fig. 3B). *S. oneidensis ryhB* also depends on a Rho-independent terminator for transcription termination, a feature shared by those of *E. coli* and *V. cholerae*.

As the transcript of *S. oneidensis ryhB* is unique in length, we intended to determine whether the well-characterized ones of different lengths, such as *ryhB*s of *E. coli* and *V. cholerae*, could serve as a functional replacement in *S. oneidensis*. By using the same expression strategy and Northern blotting validation (Fig. S2A), we found that *Ec*RyhB behaved the same as *So*RyhB, but *Vc*RyhB could not complement the *So*RyhB loss (Fig. 3B). Then, the sequences that differ between *So*RyhB and *Vc*RyhB were swapped, and the results showed that these chimeras were stable, and the upstream region (*Vc*RyhB*^So^*^-up^) but not the downstream region (*Vc*RyhB*^So^*^-down^) appeared important for this sRNA to function in *S. oneidensis* (Fig. 3A and B; Fig. S2B). Similarly, the replacement of the downstream region of *So*RyhB with the *Ec*RyhB counterpart (RyhB*^Ec^*^-down^) did not affect the RyhB activity (Fig. 3B). It was worth mentioning that the structures of *So*RyhB, *Ec*RyhB, and *Vc*RyhB predicted with multiple programs, including ViennaRNA, RNAstructure, and AlphaFold3 (23–25), were not satisfactory, with only the Rho-independent terminators being reasonably accurate (Fig. S3).

### RyhB loss reduces intracellular concentrations of labile iron

**Fig 4:**
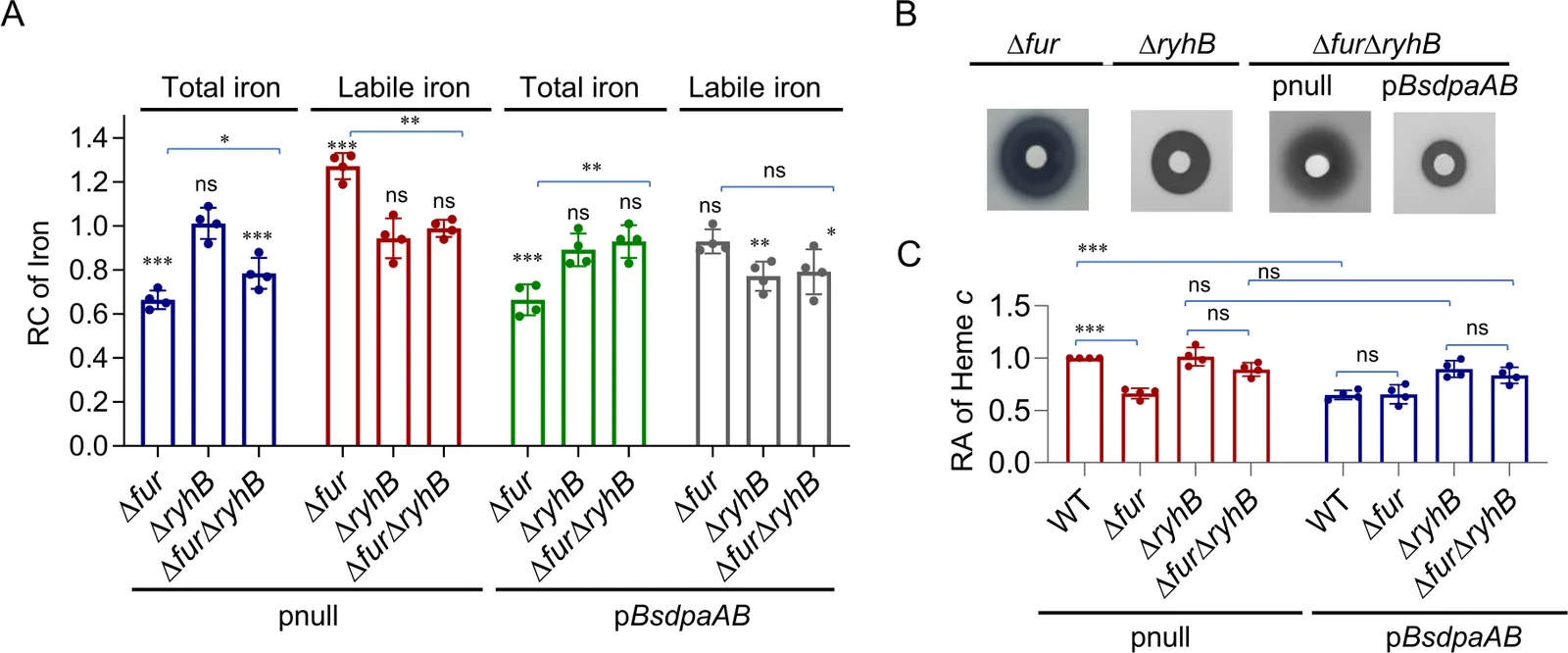
Impacts of the loss of Fur and RyhB on total and labile iron levels. (**A**) Effects of Fur and RyhB on cellular iron levels. Cultures of the strains under test entering the stationary phase were collected, and their total iron and labile iron contents were determined. The averaged levels in each strain were normalized to those of the WT, giving relative concentrations (RCs). p*BsdpaAB*, *Bacillus subtilis dpaAB* genes under the control of P*tac* with 0.2 mM IPTG. (**B**) Impacts of endogenous iron chelator on susceptibility of the indicated strains to streptonigrin by zone inhibition assays. Ten microliters of 2 mg/mL streptonigrin was added to filter paper discs (6 mm, white spot in the center) on lawns (light color area) of the indicated strains. The black area represents the inhibition zone. Plates were incubated at 30°C. (**C**) The cyt *c* contents in the strains under test grown to the early stationary phase. *B. subtilis dpaAB* genes were expressed under the control of P*tac* with 0.2 mM IPTG. In all panels, experiments were independently performed at least four times. In panels A and C, all the data are presented, and in panel B, representative data are presented. Statistical analysis was performed against the WT data in panel A and between indicated strains in panel C: ns, not significant; *, *P* < 0.05; **, *P* < 0.01; and ***, *P* < 0.001.

The Fur loss in *S. oneidensis* results in disruption of iron homeostasis, with lowered total iron content but elevated labile iron concentrations (15, 17) (Fig. 4A). To unravel how the RyhB loss suppresses the lowered cyt *c* content of the *fur* mutant, we compared iron levels of Δ*fur*, Δ*ryhB*, and Δ*fur*Δ*ryhB* to those of the WT by using a ferrozine-based colorimetric assay and electron paramagnetic resonance (EPR) spectroscopy to quantify total cellular iron and labile iron, respectively. In cells grown in LB, the impacts of the RyhB loss from the WT background on both total and labile iron levels were found to be insignificant (Fig. 4A). In contrast, the *ryhB* removal counteracted the effect of the Fur loss in both accounts: elevating and reducing the total and labile iron levels, respectively (Fig. 4A). In addition, labile iron levels in these strains were assayed with streptonigrin, a redox cycling antibiotic whose antibacterial activity correlates with the levels of intracellular free iron. The results showed that the Δ*fur*Δ*ryhB* strain, along with Δ*ryhB*, exhibited resistance to streptonigrin comparable to the WT (Fig. 4B), supporting the conclusion that the *ryhB* mutation suppresses the high labile iron phenomenon of the *fur* mutant.

The lowered cyt *c* content has been attributed to the elevated labile iron levels in the *fur* mutant (17). To determine whether the suppressing effect of the *ryhB* loss on labile iron concentrations dictates the normal cyt *c* content in Δ*fur*Δ*ryhB*, we produced dipicolinate endogenously in *S. oneidensis*, an iron chelator that is unable to cross the membranes and therefore could substantially reduce intracellular labile iron levels (26). Dipicolinate can be synthesized by *Bacillus subtilis* dipicolinate synthetase (encoded by *BsdpaAB*) through a one-step reaction from dihydrodipicolinate, an intermediate in the pathway of diaminopimelate and lysine biosynthesis (27)(Fig. S4). When *BsdpaAB* was expressed with 0.1 mM IPTG, the labile iron levels in the Δ*fur* strain were reduced to a level comparable to the averaged level in the WT (Fig. 4A), and the detrimental effect of Fur loss on cyt *c* content was no longer significant (Fig. 4C). Similarly, the expression of *BsdpaAB* lowered the labile iron levels in the WT, Δ*ryhB*, and Δ*fur*Δ*ryhB* strains (Fig. 4A). However, the cyt *c* content of the WT but not Δ*ryhB* or Δ*fur*Δ*ryhB* decreased (Fig. 4C). Altogether, these data suggest that the reduction in labile iron levels caused by RyhB loss is unlikely a decisive factor accounting for regulation of cyt *c* content by RyhB.

### Impacts of RyhB on the cyt *c* content may not be critically determined by direct regulation of cyt *c* genes

To identify the factors through which RyhB influences the cyt *c* content of *S. oneidensis*, we combined omics analyses for differentially expressed genes and *in silico* prediction for RyhB targets. By comparing the proteomics data from our previous study to the transcriptomics data from others (17, 18), we found that the impacts of RyhB loss on the proteome of the Fur regulon were much more drastic (Table 1), which is in line with the regulation of RyhB occurring post-transcriptionally.

**TABLE 1:** Expression differences of some Fur regulon members^*a*^ and cyt *c* proteins in transcriptomics and proteomics

Locus	Gene	Annotated function	Fold change		Fur regulon	RyhB regulon
			Transcriptomics^*b*^	Proteomics^*c*^		
SO_0139	*ftn*	Ferritin Ftn	ns	0.623	Y^*d*^	
SO_0264	*scyA*	Periplasmic monoheme cytochrome c5	6.54	3.623		
SO_0610	*petC*	Ubiquinol-cytochrome c reductase subunit	1.84	1.435		Y
SO_0744	*fbpA*	ABC-type Fe3+ uptake system substrate-binding component	ns	0.490	Y	
SO_0798	SO_0798	TonB-dependent receptor	ns	0.268	Y	
SO_1111	*brf2*	Bacterioferritin subunit *2* Bfr2	0.9	nd	Y	
SO_1188	SO_1188	Inner membrane protein with PepSY TM helix	ns	0.29	Y	
SO_1427	*dmsE*	Periplasmic decaheme cytochrome c	ns	nd	Y	
SO_1482	SO_1482	TonB-dependent receptor	ns	0.28	Y	
SO_1580	SO_1580	TonB-dependent heme/hemoglobin receptor	ns	0.24	Y	
SO_1755	SO_1755	Phosphoglucomutase/phosphomannomutase family protein	ns	0.47	Y	
SO_1777	*mtrA*	Decaheme cytochrome c	1.51	2.29		
SO_1778	*mtrC*	Decaheme cytochrome c	1.58	3.28		
SO_1779	*omcA*	Decaheme cytochrome c	1.42	4.05		
SO_1782	*mtrD*	Decaheme cytochrome c component MtrD	ns	nd	Y	
SO_2366	SO_2366	Two-component system response regulator	ns	nd	Y	
SO_2426	*ssoR*	Two-component signal transduction system response regulator	ns	0.23	Y	
SO_2727	*cctA*	Periplasmic tetraheme cytochrome c	3.68	2.86		Y
SO_3406	SO_3406	Putative siderophore transporter component 1	ns	0.40	Y	
SO_3420	SO_3420	Monoheme cytochrome c	2.33	2.14		
SO_3669	*hmuA*	TonB-dependent heme/hemoglobin receptor HmuA	ns	0.23	Y	
SO_3670	*tonB*	Heme uptake energy transducer component TonB	ns	0.20	Y	
SO_3914	SO_3914	TonB-dependent siderophore receptor	ns	0.22	Y	
SO_3980	*nrfA*	Cytochrome c nitrite reductase	2.69	1.64		
SO_4047	*tsdA*	SoxA-like diheme cytochrome c	2.85	2.04		
SO_4048	*tsdB*	Diheme cytochrome c4	ns	2.09		
SO_4516	SO_4516	TonB-dependent siderophore receptor	ns	0.25	Y	
SO_4523	*irgA*	Iron-responsive TonB-dependent enterobactin receptor IrgA	ns	0.20	Y	
SO_4591	*cymA*	Tetraheme cytochrome c	2.64	1.79		
SO_4666	*cytcB*	Diheme cytochrome c4	1.88	1.93		

^*a*^ The Fur regulon members that are expressed significantly differently in proteomics.

^*b*^ From reference 18. ns, not significant.

^*c*^ From reference 17. nd, no data.

^*d*^ Y, member of the regulon.

**Fig 5:**
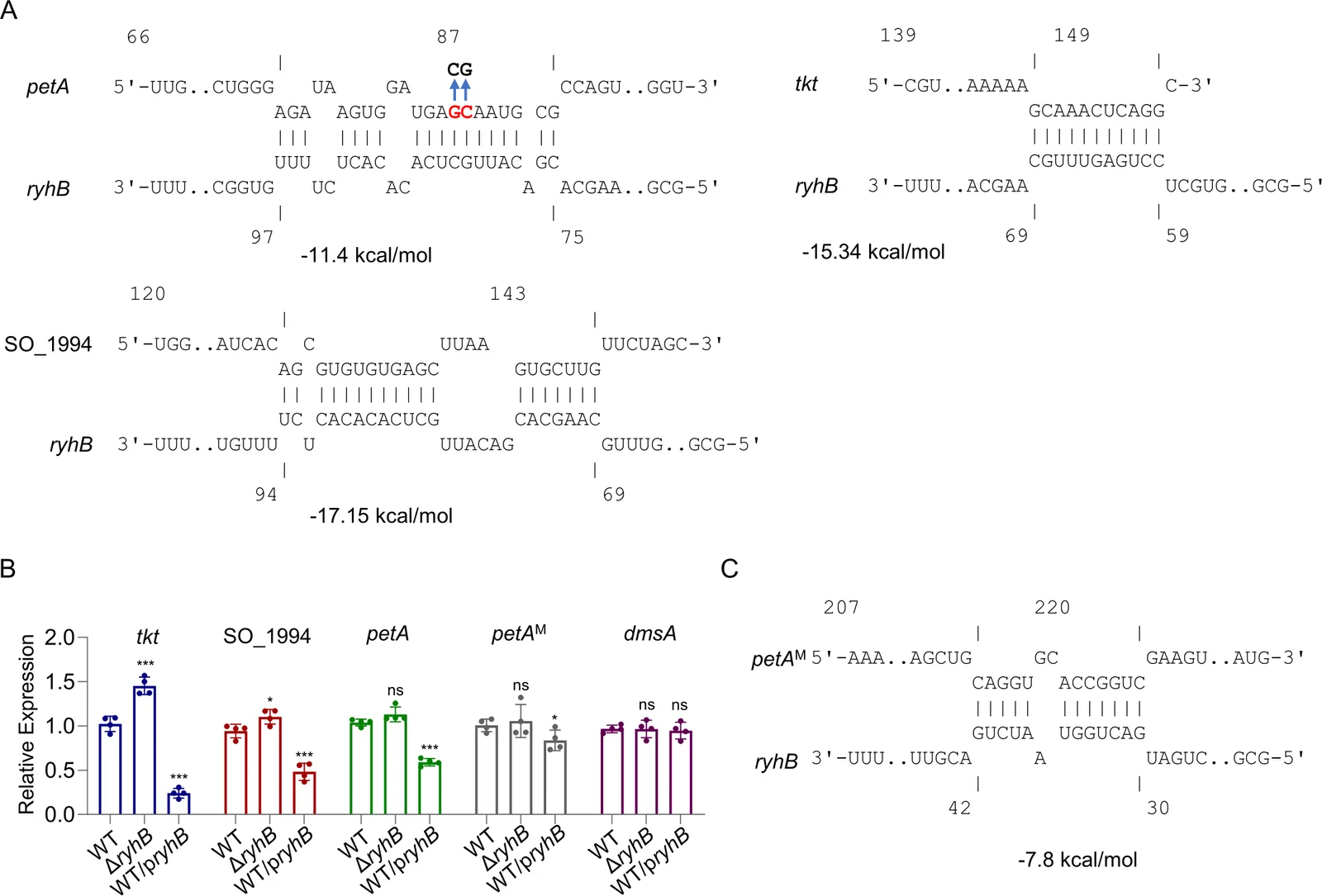
Validation of *S. oneidensis* RyhB regulon members. (**A**) Predicted pairing between RyhB sRNA and mRNAs of *petA*, *tkt*, and SO_1994. The numbers are relative to the promoter for each gene being 1. The mutation of GC to CG in *petA* is indicated. Binding energy was obtained from IntaRNA 2.0. (**B**) Expression of the gene under test reflected by its promoter activity in the indicated strains under different iron conditions by an integrative LacZ reporter. Expression of *ryhB* was induced with 0.5 mM IPTG. The averaged levels of the promoters in each strain were normalized to those of the WT (set to 1), giving a relative expression. The data from four replicates are presented as the mean ± SD. Statistical analysis was performed against the average of the WT samples: ns, not significant; *, *P* < 0.05; **, *P* < 0.01; and ***, *P* < 0.001. (**C**) Predicted pairing between RyhB sRNA and mRNAs of a *petA* mutant (*petA*^M^).

A large portion of the Fur regulon, whose transcription was hardly unaffected, was found to be expressed significantly differently in the proteomics data. Interestingly, the absence of RyhB showed opposite effects on protein levels of the cyt *c* and non-cyt *c* members of the Fur regulon, improving the former but inhibiting the latter (Table 1) (Fig. 5A). As the synthesis of cyt *c* proteins requires a post-translational modification process catalyzed by the nine-component cyt *c* maturation (CCM) system in *S. oneidensis* (28, 29), we examined whether RyhB regulates the expression of the system (Fig. S5A). Unfortunately, only CcmA, which is soluble in the cytoplasm, was detected by the proteomics analysis (Fig. S5B). To this end, the activity of all promoters for the CCM operons was monitored with the integrative LacZ reporter in WT, Δ*ryhB*, and WT overexpressing *ryhB*. The results revealed that none of the CCM operons were responsive to the absence or excess of RyhB (Fig. S5C), suggesting that CCM is unlikely to be accountable for the altered abundance of many cyts *c* linked to RyhB. Thus, it appears that RyhB employs a specific mechanism to modulate the abundance of cyts *c* but not other Fur regulon members.

RyhB regulon was then predicted with multiple *in silico* tools, including RNAPredator, CopraRNA, and RNAtarget3 (29, 30). Among the potential RyhB targets obtained, those identified by at least two programs were further analyzed with IntaRNA 2.0 (Table S1). Influence of the RyhB loss and overproduction on expression of three highly confident (based on binding energy) targets were assayed with the integrative LacZ reporter, including *petA* (SO_0608), *tkt* (SO_0930), and SO_1994 (Fig. 5B). While the non-target control gene (*dmsA*) exhibited RyhB-independent expression, all three predicted targets appeared to be subjected to regulation by RyhB, albeit regulation strength varying (Fig. 5B). For confirmation, we mutated the GC within the sequence of *petA* that interacts with RyhB to CG, presumably disrupting the projected interaction according to IntaRNA 2.0 prediction (Fig. 5C). The mutation significantly compromised the RyhB regulation (Fig. 5B). Thus, the genes on the list of RyhB targets (Table S1), at least those with high binding energy, may be directly controlled by RyhB. However, the list contains only three genes for cyt *c* proteins, SO_1421, SO_2727, and CoxB (SO_4706), supporting that direct regulation is unlikely to be the main factor underlying the increased cyt *c* content caused by RyhB.

### RyhB loss results in enhanced heme biosynthesis and reduced heme uptake/degradation

**Fig 6:**
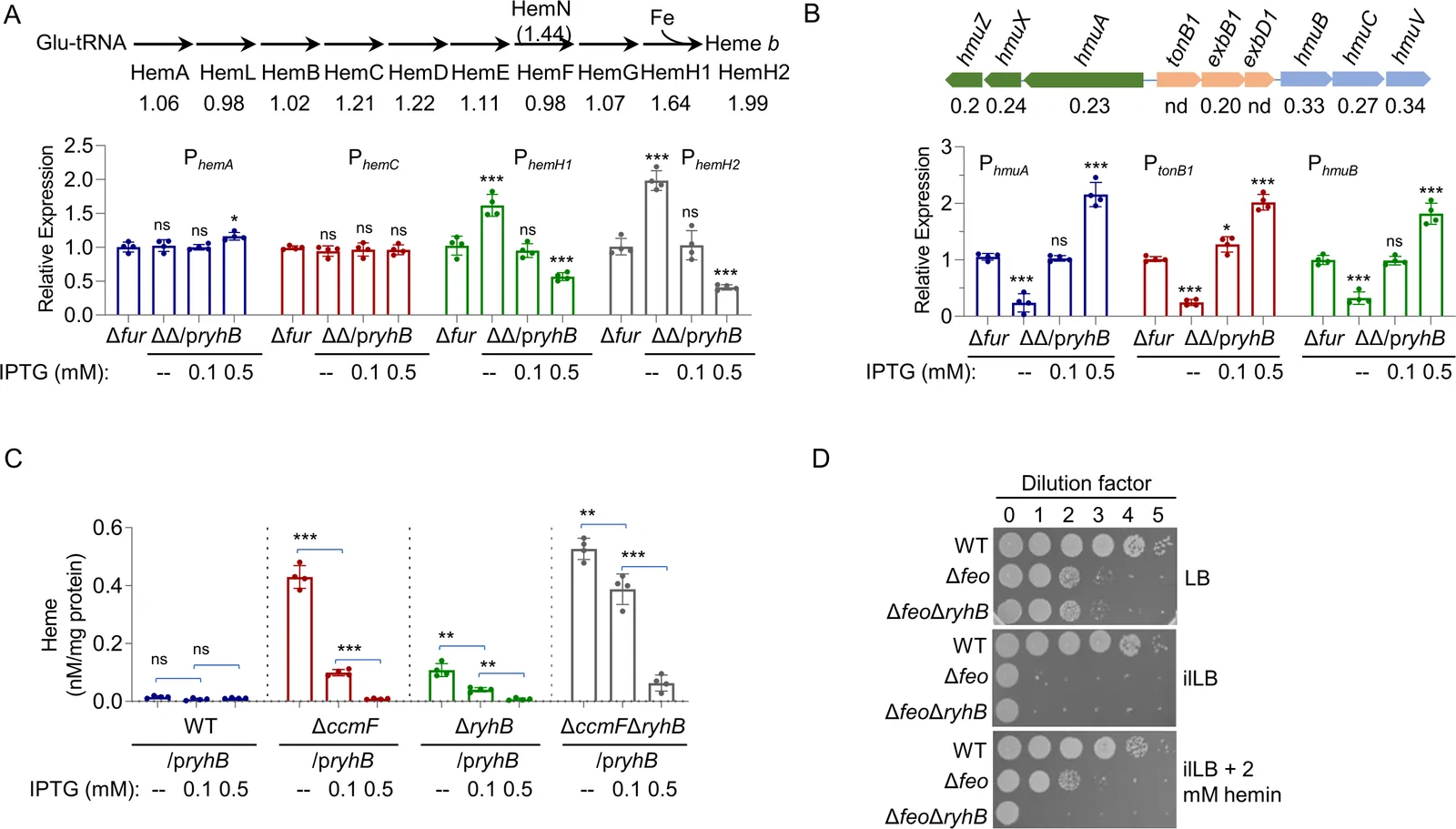
RyhB homeostasis associates with heme biosynthesis and degradation. (**A**) Expression of key heme biosynthetic genes in Δ*fur* and Δ*fur*Δ*ryhB* (ΔΔ)/p*ryhB*. Pathways for heme biosynthesis in *S. oneidensis* are shown with the proteomics values (Δ*fur*Δ*ryhB* to Δ*fur*). (**B**) Expression of key heme uptake/degradation genes in Δ*fur* and Δ*fur*Δ*ryhB*(ΔΔ)/p*ryhB*. Pathways for heme uptake/degradation in *S. oneidensis* are shown with the proteomics values (Δ*fur*Δ*ryhB* to Δ*fur*). HmuZ, heme oxygenase; HmuX, heme chaperone; HmuA, TonB-dependent receptor; TonB1-ExbB1-ExbD1, TonB energy transduction system; and HmuB-HmuC-HmuD, heme ABC transporter. (**C**) Heme quantification by pyridine hemochromogen assay. (**D**) Growth (plating efficiency) of relevant strains on LB plates. The data from four replicates are presented as the mean ± SD in panels A and C, and representative data are presented in panels B and D. Statistical analysis was performed against the average of the Δ*fur* samples: ns, not significant; *, *P* < 0.05; **, *P* < 0.01; and ***, *P* < 0.001.

To uncover the decisive mechanisms accounting for elevated cyt *c* contents in Δ*ryhB*Δ*fur* relative to Δ*fur*, we turned to heme biosynthesis, uptake, and degradation. Although the operons for these processes are not on the list of predicted RyhB targets (Table S1), they are regulated by Fur (12, 15, 17). *S. oneidensis* possesses the C5 pathway for heme synthesis (Fig. 6A), which entails nine reactions that convert glutamyl-tRNA to heme *b* (31, 32). In this pathway, the first and the last steps, catalyzed by HemA and HemH, respectively, most critically determine the biosynthesis rate (Fig. 6A). The proteomic data revealed that the abundance of most of these heme biosynthesis enzymes is not significantly different between the Δ*ryhB* and Δ*fur*Δ*ryhB* strains (Fig. 6A). However, HemH1/HemH2, both of which are functional in *Shewanella* (31, 33), were upregulated in the double mutant. Expression of multiple genes for heme biosynthesis was then assayed with the integrative LacZ reporter. Consistently, *hemH1* and *hemH2* are the only *hem* genes that were expressed at significantly elevated and lowered levels when RyhB was depleted and overproduced, respectively (Fig. 6A). In contrast to heme synthesis, all components of the heme uptake and degradation pathway, which are encoded by three operons (*hmuAXZ*, *tonB*1-*exbB*1-*exbD*1, and *hmuBCV*), were substantially downregulated in the absence of RyhB, up to fivefold, in the proteomics data, and these expression data were validated with LacZ reporters (Fig. 6B).

Simultaneous increase and decrease in the production of heme-synthesizing and degrading enzymes suggest that the RyhB loss may lead to elevated heme concentrations in the cell. To substantiate this, the intracellular heme concentrations in the WT, ∆*ryhB,* and ∆*ccmF* strains expressing *ryhB* at different levels were assessed. As reported before (10), intracellular heme contents could be confidently detected in cyt *c*-deficient strains, such as ∆*ccmF* used here as the control (10), but not in the WT with the QuantiChrom heme assay kit (Fig. 6C). The heme content in ∆*ryhB* was also found to be above the detection limit. When *ryhB* was expressed, the heme levels in ∆*ccmF* and ∆*ryhB* decreased inversely with IPTG concentrations. This phenomenon was more evident in a ∆*ccmF*∆*ryhB* double knockout (Fig. 6C).

Moreover, given that expression of all the operons for the heme uptake/degradation system is severely downregulated upon the RyhB loss (Fig. 6B), we reasoned that the ability of the Δ*ryhB* strain to take up heme is likely compromised. To test this notion, we made use of a strain lacking Feo, which serves as the primary iron uptake system (16). Growth of the Δ*feo* strain was substantially impaired on LB agar plates and much worse on ilLB; additional removal of RyhB from Δ*feo* did not make a difference (Fig. 6D). However, the Δ*feo*Δ*ryhB* strain differed from its parental Δ*feo* in that its growth could not be improved with hemin addition, supporting that the RyhB loss negatively impacts the ability of *S. oneidensis* to uptake heme from the surroundings. All these data suggest that RyhB plays a critical role in regulating heme homeostasis in *S. oneidensis*.

## DISCUSSION

Over the past 3 decades, *S. oneidensis* has become one of the most extensively studied electrochemically active bacteria, holding great potential in energy conversion and environmental bioremediation (8, 34). The foundation of this potential is respiratory versatility, which depends on a variety of iron-containing proteins, cyt *c* proteins in particular (32). However, the unusually high intracellular iron content poses a great challenge to the cell, making *S. oneidensis* hypersensitive to reactive oxygen species and radiation compared to *E. coli* (35, 36). Not surprisingly, iron homeostasis and its regulation have always been a research focus in *S. oneidensis*, with Fur and RyhB being among the first regulators studied (37, 38). Despite this, the understanding of the regulation of iron homeostasis by these regulators remains obscure. In this study, we attempted to further explore how these two regulators, individually and, more importantly, through their interplay, mediate the expression of cyt *c* genes either directly or indirectly by affecting iron homeostasis.

In *S. oneidensis*, cyt *c* proteins play a central role in extracellular electron transfer and have been extensively investigated for their biochemical, structural, electrochemical, and physiological properties (8, 34). Because of evident changes in the signature color of *S. oneidensis* colonies/pellets caused by the removal and/or overproduction of Fur, RyhB, and Hfq, they were immediately linked to regulation of cyt *c* content (18, 19, 39). Two operons that include at least one cyt *c* gene (*dmsE* and *mtrD*) are under the direct control of Fur, but it is disrupted iron homeostasis that accounts for the overall lowered cyt *c* content (12, 17).

In the *E. coli* Fur-RyhB paradigm, the relationship between these two regulators is antagonistic (21). This seems to be true in *S. oneidensis*, at least with respect to the expression of cyt *c* proteins, as the loss of RyhB largely suppresses the lowered cyt *c* content resulting from the *fur* deletion. By revealing that 10 cyt *c* genes are transcriptionally upregulated in the absence of RyhB and that two of them (ScyA and CctA) contain a predicted RyhB-binding site, an early study suggests that the counteracting effect of RyhB on Fur with respect to the cyt *c* content is likely due to direct regulation (18). Here, our data, on the one hand, agree that the overall elevated cyt *c* content in the RyhB-deficient strain can be ascribed to a considerable number of cyt *c* proteins produced at elevated levels. On the other hand, we propose that this is unlikely to be a result of direct regulation because the regulon of *S. oneidensis* RyhB, predicted with a combination of multiple computational tools, contains at most three cyt *c* genes. Moreover, although the removal of *ryhB* counteracts the effect of the Fur loss in terms of disrupted iron homeostasis (17), we unexpectedly found that the antagonistic effects of RyhB on Fur do not extend to the regulation of cyt *c* biosynthesis.

In bacteria, the cyt *c* content reflects an integrative consequence of the overall activity of the CCM system, the expression of a large portion of cyts *c*, and heme supply (9, 10, 40–43). Neither the expression nor the activity of the *S. oneidensis* CCM system is subject to regulation by Fur (15), and this is also true for RyhB, eliminating the possibility that the CCM system plays a significant role in accounting for the phenotype. In *S. oneidensis*, the cyt *c* content increases with the activity of cAMP-Crp, the primary regulatory system for respiration that directly activates a large portion of cyt *c* genes, but it could not be significantly affected by overexpressing a single or a few cyt *c* genes in general (28, 44). Thus, given that RyhB may interact with transcripts of three cyt c genes, the chance that RyhB influences cyt *c* content through direct regulation is low.

The increased cyt *c* content can come with elevated intracellular heme levels, as well as specifically increased heme supply to the CCM system mediated by heme-trafficking proteins (9, 17). In line with this, we demonstrated that the perceivable influence of RyhB on cyt *c* content in *S. oneidensis* is associated with changes in heme concentrations, largely by affecting heme biosynthesis in an indirect manner. Both Fur and Hfq have been implicated in the regulation of intracellular heme levels. Fur appears to achieve this by directly repressing the heme uptake/degradation pathway without significantly affecting heme biosynthesis (12, 17). Hfq also impacts the degradation system, but more importantly, the heme biosynthesis pathway, as three key genes, *hemA*, *hemH*1, and *hemH*2, are upregulated when Hfq is either absent or present in excess (20). This coincides with the regulation of *hemH*1 and *hemH*2 by RyhB, an observation suggesting that RyhB is largely dependent on Hfq for regulating heme biosynthesis (18, 45).

It should be noted that regulation of iron homeostasis in *S. oneidensis* is complex and involves genus-specific regulators, such as SsoR, which is a phosphorylation-independent orphan response regulator (13). While Fur acts as the sensor for SsoR, these two regulators together constitute a transcriptional derepression-inhibition system to regulate iron homeostasis in a combinatorial way (15). In addition to the novel regulators, Fur, Hfq, and RyhB may evolve an intricate regulatory mechanism that differs from that established in *E. coli* and other bacteria. Intriguingly, it has been suggested that Fur and Hfq are synthetically lethal in *S. oneidensis* (18), but we obtained a *fur hfq* double knockout strain in this study. In contrast, we demonstrated that Hfq and RyhB constitute a synthetically lethal pair. The work to identify the critical processes controlled by RyhB and Hfq leading to this synthetic lethality is under way.

## MATERIALS AND METHODS

### Bacterial strains, plasmids, and culture conditions

The bacterial strains and plasmids used in this study are listed in Table S2. The sequences of the primers used in this study are available upon request. *E. coli* and *S. oneidensis* were grown aerobically in lysogeny broth Lennox (LB) (Difco, Detroit, MI, USA) at 37°C and 30°C, respectively, for genetic manipulation. When appropriate, the growth medium was supplemented with the following: 2,6-diaminopimelic acid (DAP), 0.3 mM; ampicillin, 50 μg/mL; kanamycin, 50 μg/mL; gentamicin (Gm), 15 μg/mL; and streptonigrin, 2 mg/mL.

### Growth assessment

*S. oneidensis* strains were cultivated in LB or iron-limited LB, which contains 60 μM DFO, as well as a PIPES-buffered minimal salts (MS and ilMS) medium containing 30 mM lactate as an electron donor (46). As growth data from LB and MS were consistent and growth in ilMS was rather poor as previously reported (15), the data from the complex media were presented in this study. For aerobic growth, 3 mL of fresh medium was inoculated with 30 μL of overnight culture at 30°C and shaken at 250 rpm. Growth was monitored by measuring the optical density at 600 nm (OD_600_). Fresh media were inoculated with overnight cultures at a 200-fold dilution (OD_600_ ~ 0.01), shaken at 200 rpm at 30°C, and growth was measured by recording cell density at 600 nm (OD_600_). For droplet assays, cultures grown in liquid media to the mid-exponential phase (OD_600_ ≈ 0.4) were adjusted to contain approximately 10^8^ CFUs/mL, followed by 10-fold serial dilutions, and 5 μL of each dilution was spotted onto plates, which were incubated at 30°C for 18 h before being read.

### In-frame mutant construction

In-frame deletion strains were constructed using the *att*-based fusion PCR method as described previously (47). Briefly, two fragments flanking the gene of interest were amplified by PCR with primers containing *attB*, complementary, and gene-specific sequences, which were joined together by a second round of PCR. The fused fragments were introduced into plasmid pHGM01 using the Gateway BP Clonase II enzyme mix (Invitrogen), according to the manufacturer’s instructions. The resulting vectors were maintained in *E. coli* DAP auxotroph WM3064 and subsequently transferred into relevant *S. oneidensis* strains via conjugation. Integration of the deletion construct into the chromosome was selected by resistance to Gm and confirmed by PCR. Verified transconjugants were grown in NaCl-less LB and plated on LB supplemented with 10% sucrose. Gm-sensitive and sucrose-resistant colonies were screened by PCR for the intended deletions. Mutants were verified by sequencing the mutated region.

### Gene expression

Genetic complementation of all mutants used in the study was carried out with the isopropyl-β-D-thiogalactopyranoside (IPTG)-inducible gene expression vector pHGEN-P*tac* (48). The gene of interest was generated by PCR and introduced into the vector after the P*tac* promoter. After verification by sequencing, the resultant vectors were transferred into the relevant strains via conjugation. Expression of the gene of interest was induced by IPTG at varying concentrations to achieve full complementation. The same approach was utilized to examine the physiological impacts of target genes in overexpression.

### Analysis of gene expression

A single-copy integrative *lacZ* reporter system was used to assess the activity of target promoters, as described previously (49). Briefly, a fragment containing the sequence upstream of the target operon up to 400 bp (relative to the translation start codon) was amplified, cloned into the reporter vector pHGEI01, and verified by sequencing. The resultant vector was then transferred by conjugation into relevant *S. oneidensis* strains, where it integrated into the chromosome, and the antibiotic marker was subsequently removed (50). Cells grown to the proper phase were collected, and β-galactosidase activity was determined by using a Synergy 2 Pro200 multi-detection microplate reader (Tecan), presented in Miller units.

### Chemical assays

Through this study, protein concentrations were determined with a bicinchoninic acid assay kit with bovine serum albumin as a standard according to the manufacturer’s instructions (Pierce Chemical). Standard curves were made with commercial agents each time, where needed.

#### Iron

Total iron content was determined using the ferrozine assay (46). Cells entering the stationary phase were collected, washed with phosphate-buffered saline (PBS), adjusted to similar densities (~0.8 of OD_600_) in PBS, and sonicated. The cell lysates (100 µL) were mixed with 100 µL of 10 mM HCl and 100 µL of iron-releasing reagent (a freshly mixed solution of equal volumes of 1.4 M HCl and 4.5% [wt/vol] KMnO_4_) and treated at 60°C for 2 h. After cooling, the iron detection reagents were added to the reaction mixture (final concentrations: 20.3 mM ferrozine, 100 mM potassium acetate, and 50 mM ascorbic acid). The absorbance of samples was measured at 562 nm 140 min later. Quantification of free iron was carried out by EPR spectroscopy essentially as previously described (46). Concentrations of intracellular free iron were also assessed by measuring streptonigrin susceptibility with disk diffusion assays.

#### Heme *b* and *c*

Cultures of *S. oneidensis* strains grown in LB to the early stationary phase were aliquoted to contain similar numbers of cells and centrifuged. The resulting pellets were photographed and subsequently subjected to heme quantification. The whole bacterial proteome was extracted using the Bacterial Protein Extraction Kit (Sangon Biotech, Shanghai, China), and the total heme *b* content in *S. oneidensis* cells was determined with the QuantiChrom heme assay kit as described before (10). To quantify heme *c* levels, the extracted proteome was subjected to denaturation by trichloroacetic acid precipitation to release un-covalently attached heme, and the precipitated protein portion was used for heme *c* measurement (9).

### SDS-PAGE and heme staining

Unless otherwise noted, cells of the late exponential phase were harvested, washed with PBS, resuspended in the same buffer, and sonicated. For heme staining, the cell lysates were separated on SDS-PAGE using 12% polyacrylamide gels and stained with 3,3′,5,5′-tetramethylbenzidine as described elsewhere (40).

### Northern blotting and 5′-RACE analysis of transcripts

Cultures of *S. oneidensis* grown to the mid-exponential phase were harvested, and total RNA was extracted with RNeasy Kit (Qiagen), following the instructions of the manufacturer. Total RNA was separated on 1% (wt/vol) agarose gels. Gene-specific digoxigenin (DIG) probes were PCR generated by using a PCR DIG probe synthesis kit (Roche Diagnostics). Blotting, hybridization, and washing conditions were conducted according to the protocol described elsewhere (51). The transcriptional start site of the *ryhB* gene of *S. oneidensis* was determined using a 5′-RACE kit (Invitrogen) as described before (52). Reverse transcription was conducted on preprocessed RNAs without 5′-phosphates, followed by two rounds of nested PCRs. The PCR products were subjected to agarose gel separation, with purification of the 5′-RACE products, and inserted into the pMD19-T vector (TaKaRa) for direct DNA sequencing. The first DNA base adjacent to the 5′-RACE adaptor was regarded as the transcription start site.

### Sequencing, proteomics, bioinformatics, and statistical analyses

Sequencing and identification of mutations, including insertions and deletions, were done by BGI (BGI, Shenzhen, China) with the reference genome sequence obtained from GenBank (NC_004347.2). The proteomics data were from the ProteomeXchange Consortium (http://proteomecentral.proteomexchange.org) (data set ID PXD021732). Promoters were predicted with iPro70-FMWin (53). Student’s *t* test was performed for pairwise comparisons. Values were presented as means ± standard error of the mean.

## Data Availability

All data associated with this work are included in the paper and the supplemental material.

## SUPPLEMENTAL MATERIAL

Supplemental material aem.01500-26-s0001.pdf Fig. S1 to S5; Tables S1 and S2.

ASM does not own the copyrights to Supplemental Material that may be linked to, or accessed through, an article. The authors have granted ASM a non-exclusive, world-wide license to publish the Supplemental Material files. Please contact the corresponding author directly for reuse.
