# Supplementary material for: Effects of RyhB on Fur regulation of cytochrome c synthesis in Shewanella oneidensis
Source: Appl Environ Microbiol. 2026 Sep 30;92(10):e01500-26. doi: 10.1128/aem.01500-26 (PMC13688836; doi:10.1128/aem.01500-26)
Supplement: Supplementary file 1 [file aem.01500-26-s0001.pdf]

Supporting Information for

The effects of RyhB on Fur regulation of cytochrome *c* synthesis in *Shewanella oneidensis*

Kaiyue Jie,[1 #] Xinyue Liu,[1#] Fuqian Zhang,[1] Jim Yah,[1] Lulu Liu, [1,2]* and Haichun Gao[1]*

*Corresponding author:

Lulu Liu, lulu.liu@path.ox.ac.uk

Haichun Gao, haichung@zju.edu.cn

This PDF file includes:

Supplementary Figures S1 to S5

Supplementary Tables S1 to S2

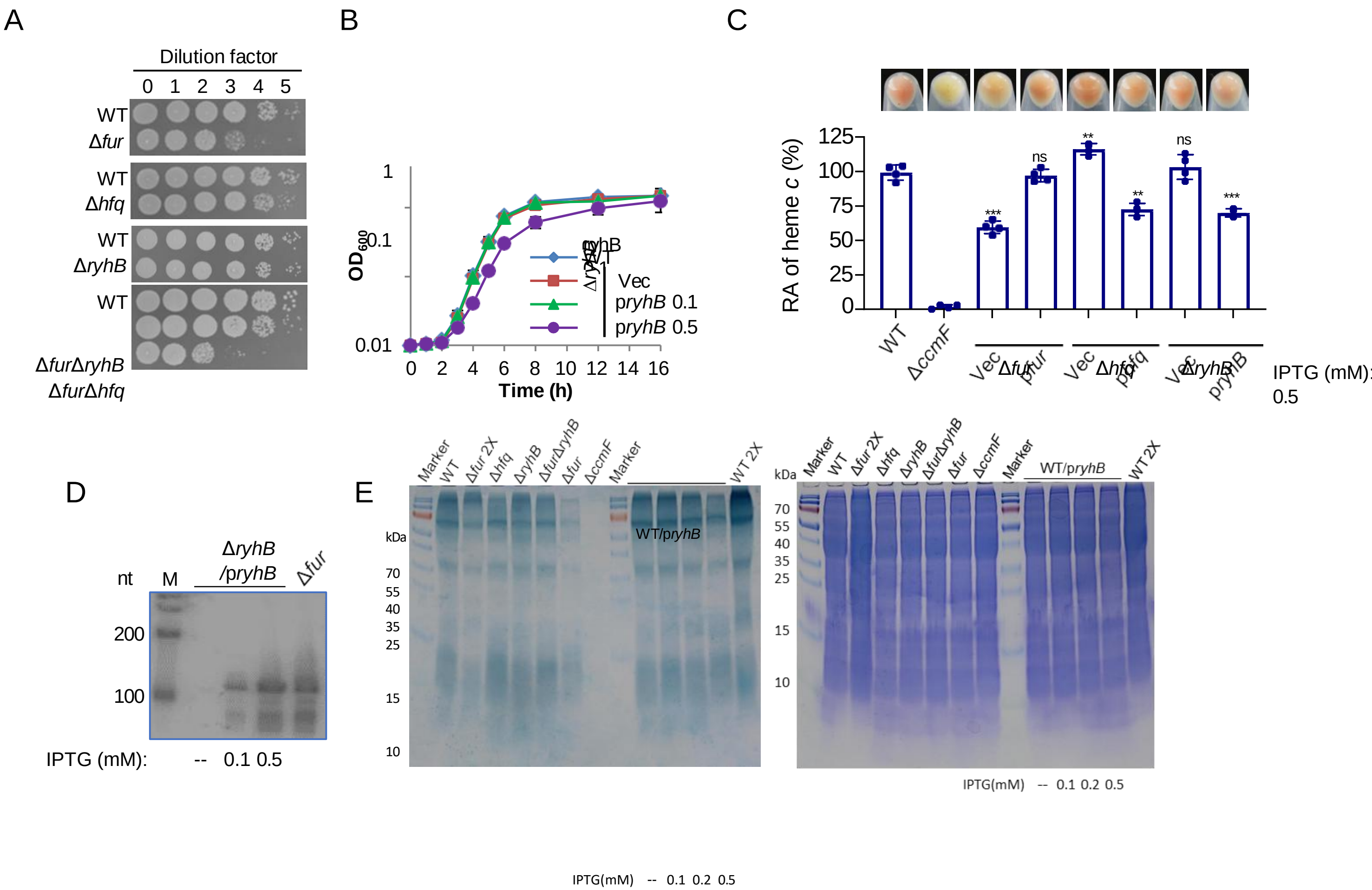

FIG S1. The physiological impacts of the losses of Fur, Hfq, and RyhB in *S. oneidensis*. (A) Growth of the indicated strains on LB agar plates. (B) Growth of the indicated strains in LB. p*ryhB*, expressing a copy of *ryhB* on plasmid *in trans* for complementation. The gene was under the control of IPTG-inducible promoter P*tac*. (C) Cell pellets and cyt *c* levels in the strains under test. The heme *c* levels of the WT and cytochrome *c* deficient strain Δ*ccmF* were set to 1 and 0, respectively. (D) Northern blotting analysis of RyhB expression. The cultures from B were collected for Northern blotting assay. (E) Heme staining of the indicated strains. After SDS-PAGE separation, cyts *c* were specifically stained. Samples were from Fig. 1B and Fig. S1C. On the right, the duplicated gel was stained with Coomassie brilliant blue as the loading control. 2X, double loading.

In A and B, the data of four replicates were presented as the mean ± SD. Statistics analysis was performed against the average of the WT samples: ns, not significant; *. $p < 0.05$; **, $p < 0.01$; ***, $p < 0.001$.

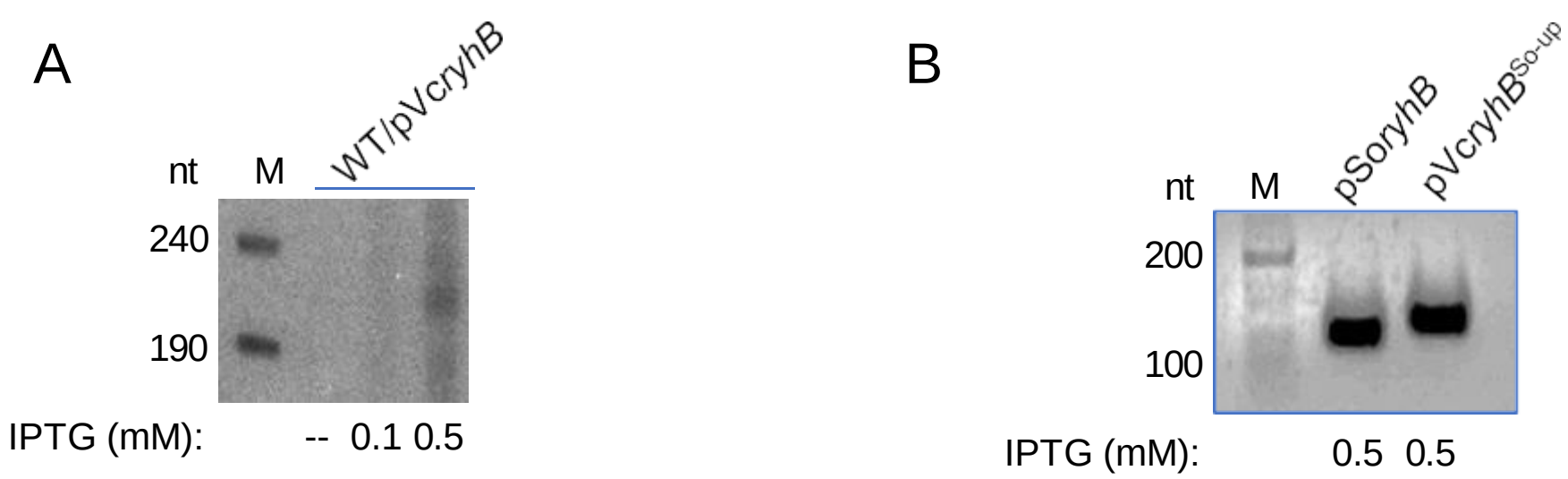

FIG S2. Stability of RyhB chimeras. Northern blotting analysis of representative RyhB chimeras.

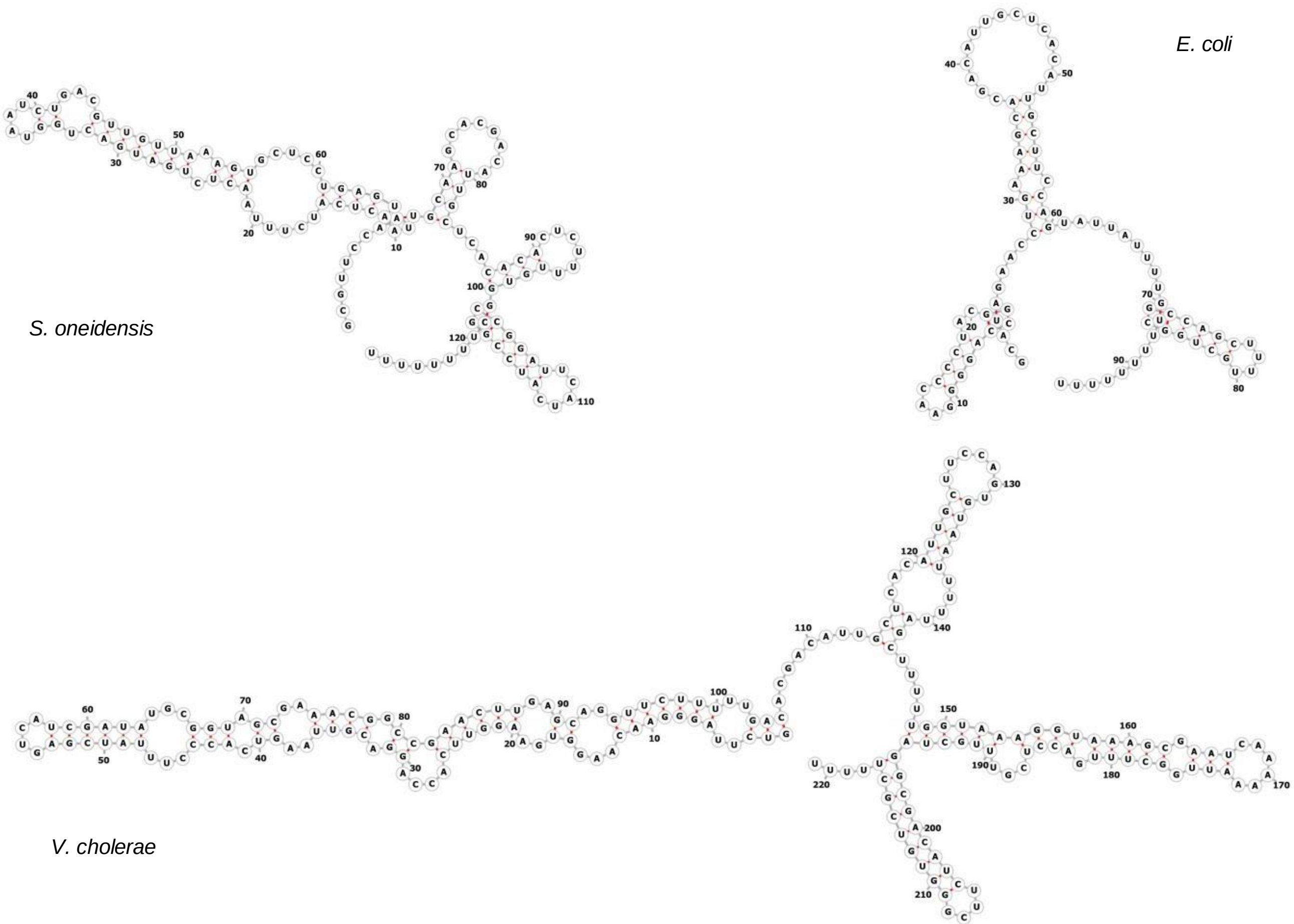

FIG S3. Predicted structure of *E. coli*, *S. oneidensis*, *V. cholerae* RyhBs. Multiple programs were used but the resulting structures exhibited low consistency. Shown were the structures from ViennaRNA as the structure of *Ec*RyhB is similar to those reported before.

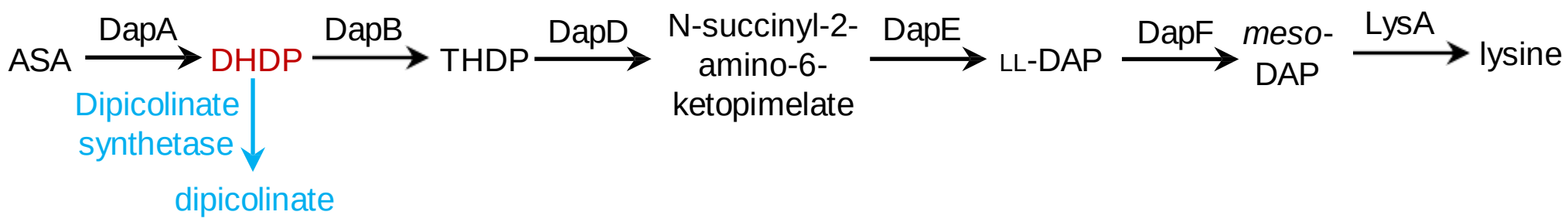

FIG S4. Pathway of diaminopimelate and lysine biosynthesis. The conversion of dihydrodipicolinate (DHDP) to dipicolinate by *B. subtilis* dipicolinate synthase was shown in blue. ASA, aspartate semialdehyde; THDP, tetrahydrodipicolinate, LL-DAP, L,L-diaminopimelate.

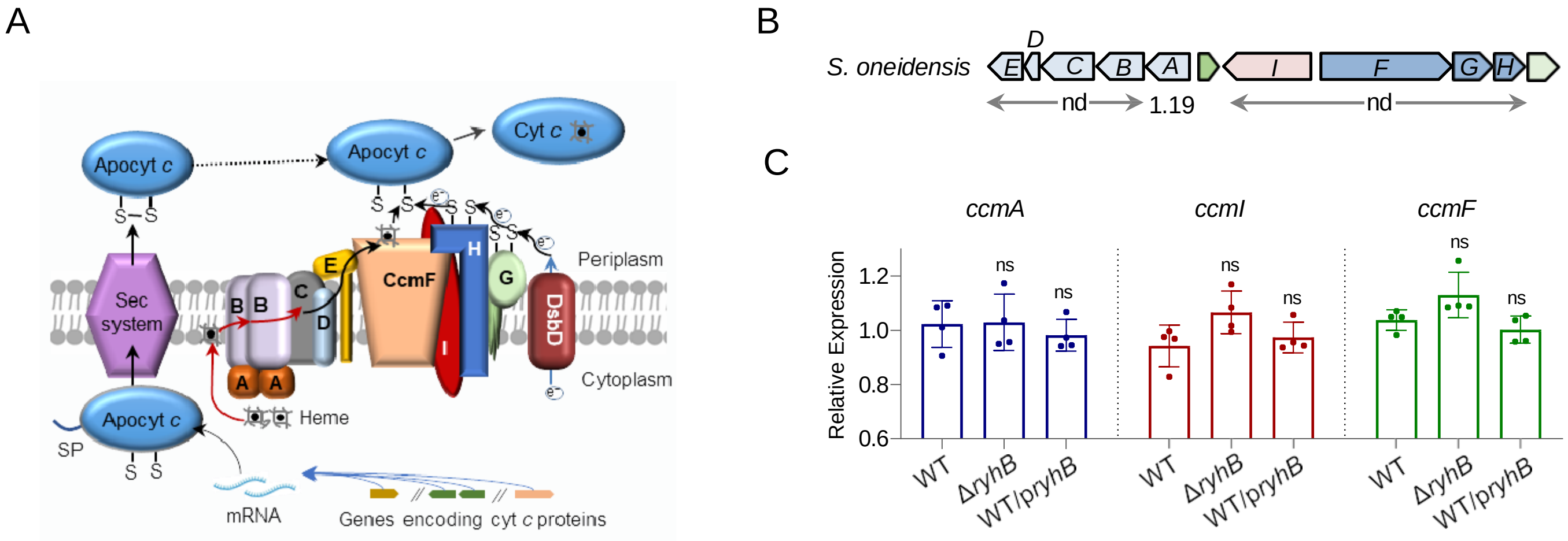

FIG S5. Expression of the cyt c maturation system is independent of RyhB. (A) Schematic overview of cyt *c* maturation. After transcription and translation, cyt *c* genes produce apocyt *c* proteins, which enters the periplasm via the Sec system. These apocyt *c* proteins have to go through oxidation first, and then reduction by using electrons mediated CcmGH(I) and DsbD, which gets electrons from the cytoplasm. Heme ligation to reduced apocyt *c* proteins is catalyzed by CcmF after receiving heme from CcmABCDE. (B) The operons for the cyt *c* maturation system. The genes encoding cyt *c* maturation components from the same operon were shown in the same color. Proteomics values were shown. (C) Expression of the genes under test reflected by its promoter activity in indicated strains by an integrative LacZ reporter. The averaged levels of the promoters in each strain were normalized to those of the WT (set to 1), giving to relative expression. Expression of *ryhB* was induced with 0.5 mM IPTG. The data of four replicates were presented as the mean ± SD. Statistics analysis was performed against the average of the WT samples: ns, not significant; *. $p < 0.05$; **, $p < 0.01$; ***, $p < 0.001$.

Table S1 Potential targets of RyhB predicted by at least two of three tools, RNAPredator, CopraRNA, and RNAtarget3[a]

Target	Locus	Start	End	Energy	p-value	gene	annotation
SO_RS00005	SO_0001	61	89	-11.53	0.0133172	mioC	FMN-binding protein MioC
SO_RS00010	SO_0002	136	148	-10.21	0.024771		POT family MFS transporter
SO_RS00035	SO_0007	49	80	-10.84	0.0208496	rpmH	50S ribosomal protein L34
SO_RS00090	SO_0018	124	150	-14.33	0.0017421		MOSC domain-containing protein
SO_RS00205	SO_0041	3	26	-10.67	0.0232166		DUF1488 domain-containing protein
SO_RS00330	SO_0065	106	119	-10.65	0.0235105	mog	molybdopterin adenylyltransferase
SO_RS00550	SO_0112	58	78	-11.97	0.0099048		MliC family protein
SO_RS00590	SO_0121	1	46	-11.13	0.0173096		mechanosensitive ion channel
SO_RS00725	SO_0154	31	54	-10.57	0.0247195		FUSC family protein
SO_RS00775	SO_0168	8	27	-12.53	0.0075488	gspF	type II secretion system inner membrane protein GspF
SO_RS01415	SO_0293	51	79	-10.58	0.0205443		phosphoglycolate phosphatase
SO_RS01420	SO_0294	61	73	-10.98	0.0167637	trpS	tryptophan--tRNA ligase
SO_RS01910	SO_0397	1	9	-10.85	0.0207174		fumarate reductase cytochrome b subunit
SO_RS01980	SO_0410	1	43	-10.53	0.0253448	mutT	8-oxo-dGTP diphosphatase MutT
SO_RS02180	SO_0454	2	10	-10.71	0.0226389		DUF1850 domain-containing protein
SO_RS02185	SO_0455	56	88	-11.43	0.0133182		TRAP transporter permease
SO_RS02890	SO_0608	66	87	-11.4	0.0145121	petA	ubiquinol-cytochrome c reductase iron-sulfur subunit
SO_RS02960	SO_0622	59	70	-10.59	0.0244121		response regulator transcription factor
SO_RS03025	SO_0640	65	89	-14.63	0.0013687		NAD(P)H-quinone oxidoreductase
SO_RS03245	SO_0683	63	91	-11.43	0.0142281		Derived by automated computational analysis using gene prediction method: GeneMarkS-2+.
SO_RS03465	SO_0735	131	150	-10.65	0.0235105		Derived by automated computational analysis using gene prediction method: Protein Homology.
SO_RS03560	SO_0756	63	86	-12.84	0.0053821	aroG	3-deoxy-7-phosphoheptulonate synthase AroG
SO_RS03840	SO_0816	23	30	-11.7	0.0115936		HDOD domain-containing protein
SO_RS04365	SO_0930	139	149	-15.34	0.0007573	tkt	transketolase
SO_RS04405	SO_0938	106	112	-11.53	0.0133172		lipoprotein
SO_RS04565	SO_0973	91	105	-12.61	0.0063446		Derived by automated computational analysis using gene prediction method: Protein Homology.
SO_RS04685	SO_1003	105	125	-12.35	0.0076196		Derived by automated computational analysis using gene prediction method: Protein Homology.
SO_RS04705	SO_1008	67	97	-14.47	0.0015576		IS982-like element ISSod20 family transposase
SO_RS04755	SO_1018	83	122	-10.97	0.019189	nuoE	NADH-quinone oxidoreductase subunit NuoE
SO_RS04785	SO_1027	118	132	-11.3	0.0154965		Derived by automated computational analysis using gene prediction method: Protein Homology.
SO_RS04910	SO_1055	55	77	-12.71	0.0059083		GGDEF domain-containing protein
SO_RS04925	SO_1060	109	129	-10.85	0.0207174	lpoB	penicillin-binding protein activator LpoB
SO_RS05055	SO_1093	77	114	-13.14	0.0043266		IS4-like element ISSod7 family transposase
SO_RS05155	SO_1113	36	76	-11.39	0.014608		methyl-accepting chemotaxis protein
SO_RS05265	SO_1142	1	37	-11.8	0.0111157	carB	carbamoyl-phosphate synthase large subunit
SO_RS05345	SO_1159	35	58	-11.84	0.0108192		DNA polymerase III subunit psi
SO_RS05465	SO_1183	12	74	-13.03	0.0046895		FAD-dependent monooxygenase
SO_RS05600	SO_1202	63	95	-10.98	0.0190663	rimP	ribosome maturation factor RimP

SO_RS05780	SO_1242	118	145	-10.16	0.0254033		Derived by automated computational analysis using gene prediction method: GeneMarkS-2+.
SO_RS05935	SO_1279	134	149	-11.54	0.0125873		Derived by automated computational analysis using gene prediction method: GeneMarkS-2+.
SO_RS06075	SO_1309	44	55	-10.52	0.0211786		TonB-dependent receptor
SO_RS06320	SO_1361	82	117	-17.87	6.90E-05		3-deoxy-7-phosphoheptulonate synthase
SO_RS06330	SO_1363	81	108	-10.31	0.023552	hcp	hydroxylamine reductase
SO_RS06430	SO_1383	124	140	-11.35	0.014997		DEAD/DEAH box helicase
SO_RS06440	SO_1387	45	65	-11.91	0.010405		HD domain-containing protein
SO_RS06465	SO_1392	120	140	-11.04	0.0183452		HPP family protein
SO_RS06510	SO_1402	69	87	-11.64	0.0123765		PAS domain-containing protein
SO_RS06585	SO_1417	118	143	-12.25	0.008169		PhnD/SsuA/transferrin family substrate-binding protein
SO_RS06605	SO_1421	54	78	-11.21	0.0164337		flavocytochrome c
SO_RS06915	SO_1489	82	146	-11.38	0.0147044		Derived by automated computational analysis using gene prediction method: Protein Homology.
SO_RS07080	SO_1524	68	90	-11.85	0.0107463	grpE	nucleotide exchange factor GrpE
SO_RS07305	SO_1578	50	69	-12.2	0.0084571		TetR/AcrR family transcriptional regulator
SO_RS07320	SO_1581	126	137	-10.29	0.0237911		alkylphosphonate utilization protein
SO_RS07445	SO_1612	127	137	-12.8	0.0055393		DUF3192 domain-containing protein
SO_RS07455	SO_1614	82	107	-10.31	0.023552		GNAT family N-acetyltransferase
SO_RS07835	SO_1700	21	29	-11.87	0.0106017		DUF4785 family protein
SO_RS07880	SO_1711	66	93	-12.7	0.0059507		DUF4303 domain-containing protein
SO_RS07980	SO_1738	66	108	-13.18	0.0042012		YajD family HNH nuclease
SO_RS08310	SO_1808	45	80	-15	0.0010093	pspB	envelope stress response membrane protein PspB
SO_RS08435	SO_1833	77	114	-13.14	0.0043266		IS4-like element ISSod7 family transposase
SO_RS08690	SO_1880	13	22	-10.07	0.0265809	bamC	outer membrane protein assembly factor BamC
SO_RS08695	SO_1881	28	72	-11.45	0.0140415		efflux RND transporter periplasmic adaptor subunit
SO_RS08755	SO_1893	14	24	-10.74	0.0189418		hydroxymethylglutaryl-CoA lyase
SO_RS08960	SO_1940	57	68	-12.48	0.0077473		Derived by automated computational analysis using gene prediction method: Protein Homology.
SO_RS09205	SO_1994	120	143	-17.15	0.0001438		lytic murein transglycosylase
SO_RS09260	SO_2008	65	96	-10.5	0.0213943		DUF692 domain-containing protein
SO_RS09460	SO_2054	64	81	-12.3	0.0085053		S-(hydroxymethyl)glutathione dehydrogenase/class III alcohol dehydrogenase
SO_RS09525	SO_2072	107	116	-10.95	0.017022	hisC	histidinol-phosphate transaminase
SO_RS10030	SO_2182	100	125	-11.01	0.0187026		VOC family protein
SO_RS10195	SO_2218	60	114	-10.96	0.0193123	asnS	asparagine--tRNA ligase
SO_RS10270	SO_2236	55	69	-11.08	0.0178782	crr	PTS glucose transporter subunit IIA
SO_RS10450	SO_2278	31	40	-10.74	0.0189418	ilvN	acetolactate synthase small subunit
SO_RS10535	SO_2297	49	74	-11.06	0.0160934		CPXCG motif-containing cysteine-rich protein
SO_RS10670	SO_2327	47	65	-13.95	0.0023479		chemotaxis response regulator protein-glutamate methylesterase
SO_RS10700	SO_2333	3	59	-11.53	0.0133172		alpha/beta fold hydrolase
SO_RS10705	SO_2335	100	149	-13.54	0.0032124	seqA	replication initiation negative regulator SeqA
SO_RS11660	SO_2533	49	107	-14.88	0.0011151		DUF3108 domain-containing protein
SO_RS11810	SO_2564	122	139	-10.8	0.0213861		LysM peptidoglycan-binding domain-containing protein
SO_RS12080	SO_2621	3	78	-10.78	0.021659		Nif3-like dinuclear metal center hexameric protein

SO_RS12230	SO_2654	27	39	-13.05	0.0046215		Mu transposase C-terminal domain-containing protein
SO_RS12335	SO_2673	55	81	-12.32	0.0077808		Derived by automated computational analysis using gene prediction method: GeneMarkS-2+.
SO_RS12340	SO_2674	44	88	-10.85	0.0207174		TraR/DksA family transcriptional regulator
SO_RS12590	SO_2727	75	83	-11.73	0.011416		cytochrome c3 family protein
SO_RS12825	SO_2769	50	65	-11.23	0.0162211		D-hexose-6-phosphate mutarotase
SO_RS13310	SO_2871	69	98	-15.47	0.0006773	arsC	arsenate reductase (glutaredoxin)
SO_RS13325	SO_2874	2	19	-11.07	0.0179939		IS630-like element ISSod10 family transposase
SO_RS13615	SO_2936	79	110	-10.93	0.0196868		sialidase family protein
SO_RS13775	SO_2971	121	129	-10.3	0.0236712		HP1 family phage holin
SO_RS13795	SO_4791	7	26	-11.99	0.0097705		Lambda phage conserved protein
SO_RS13855	SO_2986	63	78	-12.39	0.0074094		Derived by automated computational analysis using gene prediction method: Protein Homology.
SO_RS14010	SO_3021	137	144	-11.16	0.0152922	trpD	anthranilate phosphoribosyltransferase
SO_RS14230	SO_3067	21	44	-10.74	0.0222141	folC	bifunctional tetrahydrofolate synthase/dihydrofolate synthase
SO_RS14320	SO_3088	49	72	-11.63	0.0120185	fadJ	fatty acid oxidation complex subunit alpha FadJ
SO_RS14360	SO_3096	2	36	-13.77	0.0026972		sigma-70 family RNA polymerase sigma factor
SO_RS14505	SO_3125	7	61	-14.09	0.0021053		DEAD/DEAH box helicase
SO_RS14665	SO_3148	29	56	-10.27	0.0240325		amidohydrolase family protein
SO_RS14695	SO_3154	124	140	-11.5	0.0135847		proline--tRNA ligase
SO_RS14700	SO_3155	25	81	-11.21	0.0164337		patatin-like phospholipase family protein
SO_RS14920	SO_3198	78	106	-13.36	0.0036766		Derived by automated computational analysis using gene prediction method: Protein Homology.
SO_RS15155	SO_3248	65	81	-10.07	0.0265809	flgD	flagellar hook assembly protein FlgD
SO_RS15265	SO_3269	19	62	-11.6	0.0122053		glycosyltransferase family protein
SO_RS15375	SO_3296	104	115	-13.96	0.0035715		IS5-like element ISSod12 family transposase
SO_RS15620	SO_3350	140	150	-10.47	0.0217217		PilT/PilU family type 4a pilus ATPase
SO_RS15680	SO_3364	97	136	-11.91	0.010405		YggN family protein
SO_RS15845	SO_3396	19	30	-10.8	0.0213861		methyl-accepting chemotaxis protein
SO_RS15910	SO_3412	14	37	-11.82	0.0109665		transporter substrate-binding domain-containing protein
SO_RS16435	SO_3517	81	121	-10.64	0.0199285		NAD(P)/FAD-dependent oxidoreductase
SO_RS16560	SO_3544	77	114	-13.14	0.0043266		IS4-like element ISSod7 family transposase
SO_RS16715	SO_3577	118	136	-12.61	0.0063446	clpB	ATP-dependent chaperone ClpB
SO_RS16775	SO_3587	123	149	-11.5	0.0135847		YcxB family protein
SO_RS16825	SO_3599	113	143	-10.2	0.0248962	cysP	thiosulfate ABC transporter substrate-binding protein CysP
SO_RS17400	SO_3736	129	148	-13.02	0.0047238		phosphoadenylyl-sulfate reductase
SO_RS17465	SO_3750	117	149	-12.24	0.0082259		pitrilysin family protein
SO_RS17510	SO_3758	8	28	-16.71	0.0002202		sodium-dependent bicarbonate transport family permease
SO_RS17585	SO_3774	53	86	-14.55	0.0026142	putA	bifunctional proline dehydrogenase/L-glutamate gamma-semialdehyde dehydrogenase PutA
SO_RS17770	SO_3816	102	150	-10.64	0.0199285		VanZ family protein
SO_RS17940	SO_3854	104	115	-12.87	0.0063247		IS5 family transposase
SO_RS18015	SO_3870	60	98	-11.88	0.0105672		disulfide bond formation protein B
SO_RS18120	SO_3892	119	149	-13.56	0.0031643		superinfection exclusion B family protein
SO_RS18285	SO_3928	44	97	-13.11	0.004423	rpsR	30S ribosomal protein S18

SO_RS18330	SO_3938	13	53	-10.18	0.0251485		DUF2065 domain-containing protein
SO_RS18840	SO_4056	39	51	-12.15	0.0087543	metB	cystathionine gamma-synthase
SO_RS19160	SO_4135	77	114	-13.14	0.0043266		IS4-like element ISSod7 family transposase
SO_RS19660	SO_4240	102	144	-12.06	0.0093134		diguanylate cyclase
SO_RS19765	SO_4262	68	86	-16.16	0.0003674		Derived by automated computational analysis using gene prediction method: GeneMarkS-2+.
SO_RS20245	SO_4359	54	82	-13.58	0.004362		MtrB/PioB family decaheme-associated outer membrane protein
SO_RS20285	SO_4368	1	11	-11.3	0.0154965		phosphopantetheine-binding protein
SO_RS20315	SO_4374	130	149	-10.73	0.0223549		aromatic amino acid ammonia-lyase
SO_RS20330	SO_4377	74	93	-10.77	0.0217966		MMPL family transporter
SO_RS20505	SO_4418	24	35	-10.5	0.0213943		CHAP domain-containing protein
SO_RS20530	SO_4424	83	106	-14.31	0.00177		YqaE/Pmp3 family membrane protein
SO_RS20560	SO_4431	29	73	-11.31	0.0153954		Derived by automated computational analysis using gene prediction method: Protein Homology.
SO_RS20655	SO_4451	77	86	-10.14	0.0256605	moaC	cyclic pyranopterin monophosphate synthase MoaC
SO_RS20835	SO_4490	141	147	-11.14	0.0171978		Derived by automated computational analysis using gene prediction method: Protein Homology.
SO_RS20940	SO_4506	51	57	-11.94	0.0101093		4Fe-4S binding protein
SO_RS20955	SO_4509	60	73	-12.01	0.0096378		molybdopterin-dependent oxidoreductase
SO_RS21290	SO_4583	58	73	-10.76	0.021935	rpoH	RNA polymerase sigma factor RpoH
SO_RS21335	SO_4592	5	13	-10.89	0.0201962		Derived by automated computational analysis using gene prediction method: Protein Homology.
SO_RS21400	SO_4607	78	107	-11.51	0.013495	ctaD	cytochrome c oxidase subunit I
SO_RS21925	SO_4717	2	13	-11.8	0.0110119		cache domain-containing protein
SO_RS22635	SO_A0109	7	33	-14.97	0.0010349		EAL domain-containing protein
SO_RS22695	SO_A0120	104	115	-13.96	0.0035715		IS5-like element ISSod12 family transposase
SO_RS22715	SO_A0125	104	115	-13.41	0.0047691		transposase

a, Values are obtained from IntaRNA 2.0.

TABLE S2 Strains and plasmids used in this study

Strain or plasmid	Description	Source or reference
Strains		
E. coli		
DH5α	Host strain for routine cloning	Lab stock
WM3064	Donor strain for conjugation, Δ*dapA*	W. Metcalf, UIUC
S. oneidensis		
MR-1	Wild type	ATCC 700550
HG0266	Δ*ccmF* mutant derived from MR-1	(47)
HG0603	Δ*hfq* mutant derived from MR-1	(20)
HG1783-4	Δ*feo* derived from MR-1	(16)
HG1937	Δ*fur* mutant derived from MR-1	(54)
HG4716	Δ*ryhB* mutant derived from MR-1	This study
HG0266-4716	Δ*ccmF*Δ*ryhB* mutant derived from MR-1	This study
HG1783-4-4716	Δ*feo*Δ*ryhB* mutant derived from MR-1	This study
HG1937-4716	Δ*fur*Δ*ryhB* mutant derived from MR-1	This study
HG1937-0603	Δ*fur*Δ*hfq* mutant derived from MR-1	This study
Plasmids		
pHGM01	Ap^r Gm^r Cm^r suicide vector	(47)
pHGEN-P*tac*	IPTG-inducible P*tac* expression vector	(44)
pHGEI01	Integrative *lacZ* reporter vector	(50)
pBBR-Cre	Helper vector for antibiotic marker removal	(49)
pHGEN-P*tac-fur*	Vector for inducible expression of *hmgA*	(15)
pHGEN-P*tac-hfq*	Vector for inducible expression of *hppD*	(20)
pHGEN-P*tac-VcryhB*	Vector for inducible expression of *ryhB*	This study
pHGEN-P*tac-VcryhB*	Vector for inducible expression of Vc*ryhB*	This study
pHGEI01-P_{hemA}	For measuring P_{hemA} activity	(31)
pHGEI01-P_{hemC}	For measuring P_{hemC} activity	(20)
pHGEI01-P_{hemH1}	For measuring P_{hemH1} activity	(20)
pHGEI01-P_{hemH2}	For measuring P_{hemH2}activity	(20)
pHGEI01-P_{ccmA}	For measuring P_{ccmA} activity	(10)
pHGEI01-P_{ccmI}	For measuring P_{ccmI} activity	(27)
pHGEI01-P_{ccmF}	For measuring P_{ccmF} activity	(27)
pHGEI01-P_{hmuA}	For measuring P_{hmuA} activity	(15)
pHGEI01-P_{tonB1}	For measuring P_{tonB1} activity	(15)
pHGEI01-P_{hmuB}	For measuring P_{hmuB} activity	(15)
pHGEI01-P_{tkt}	For measuring P_{tkt} activity	This study
pHGEI01-P_{SO_1994}	For measuring P_{SO_1994} activity	This study
pHGEI01-P_{petA}	For measuring P_{petA} activity	This study
pHGEI01-P_{dmsA}	For measuring P_{dmsA} activity	This study

REFERENCE

54. Gao T, Ju L, Yin J, Gao H. 2015. Positive regulation of the *Shewanella oneidensis* OmpS38, a major porin facilitating anaerobic respiration, by Crp and Fur. Sci Rep 5:14263. https://doi.org/10.1038/srep14263
